# Editorial: Bacterial metabolites: redefining strategies to combat antimicrobial resistance

**DOI:** 10.3389/fmicb.2026.1883638

**Published:** 2026-06-15

**Authors:** Karthika Bahuleyan Arun, Muzafar A. Rather, Valério Monteiro-Neto

**Affiliations:** 1 Department of Life Sciences, CHRIST (Deemed to be University), Bangalore, India; 2 Department of Microbiology and Immunology, University of Minnesota Medical School, Minneapolis, MN, United States; 3 Center of Biological and Health Sciences, Federal University of Maranhão, São Luís, Brazil

**Keywords:** antibiotic adjuvants, antimicrobial resistance (AMR), bacterial metabolites, metabolic resensitization, natural product discovery, postbiotic therapeutics

Antimicrobial resistance (AMR) is among the defining public health threats of our time. An estimated 1.27 million deaths in 2019 were directly attributable to bacterial AMR (Murray et al., 2022), and recent forecasts suggest that more than 39 million additional deaths could occur by 2050 if current trends continue (Naghavi et al., 2024). However, the antibiotic discovery pipeline has not kept pace with the spread of resistance among ESKAPE and other clinically critical pathogens. Bacterial secondary metabolites, which have shaped nearly every class of clinically useful antibiotics, remain a productive but underexploited source of chemical and mechanistic novelty (Bérdy, 2012; Newman and Cragg, 2020). The 13 original research and review articles in this Research Topic mark a meaningful shift in how bacterial metabolites are understood, not only as antimicrobial agents but also as multifunctional modulators of bacterial physiology, resistance mechanisms, and therapeutic responses.

A first thread running through the Research Topic is the value of underexplored microbial habitats. Niu et al. characterized *Streptomyces flavimicrosus* sp. nov., a desert-derived actinobacterium from the Turpan-Hami Basin, and demonstrated that it produces thiolutin, a sulfur-containing antibiotic with strong activity against methicillin-resistant *Staphylococcus aureus* (MRSA) at a minimum inhibitory concentration of 16 μg/mL, with additional inhibition exceeding 70% at 50 μg/mL against nine plant pathogenic fungi. This study combined targeted antibacterial screening with polyphasic taxonomy and biosynthetic gene cluster analysis, linking the metabolite to its genetic blueprint. Pattapulavar et al. applied a complementary, genome-guided strategy to *Streptomyces* sp. VITGV156, integrating metabolomics, genome mining, molecular docking, and molecular dynamics simulations to identify candidate metabolites that bind QnrB1 and Tet(X4), proteins driving fluoroquinolone and tigecycline resistance, respectively. Together, these studies show how classical microbiology, combined with genomics and computational chemistry, can shorten the path from an environmental isolate to a mechanistically defined antimicrobial lead.

In addition to discovering new antimicrobial compounds, this Research Topic highlights the growing trend of utilizing bacterial metabolites as agents to modify resistance and as therapeutic adjuvants. Chigozie and Esimone reviewed how natural products can inhibit efflux pumps, neutralize β-lactamases, dismantle biofilms, and interfere with quorum sensing, and how synthetic biology, multi-omics workflows, and machine learning are reshaping the way candidate molecules are prioritized and optimized. By restoring the activity of existing antibiotics rather than replacing them, the adjuvant strategy offers a more sustainable use of an antimicrobial inventory that is unlikely to be rapidly replenished.

Several articles converge on the idea that bacterial metabolism itself is a druggable axis. Fu et al. reported that deletion of *ihfA* and *ihfB* in *Escherichia coli* lowers intracellular alanine, weakens the proton motive force, and reduces aminoglycoside susceptibility, a phenotype reversed by exogenous alanine supplementation. Two independent studies have extended this principle to *Pseudomonas aeruginosa*. Nageeb et al. found that glutamate potentiates gentamicin against clinical isolates by increasing membrane permeability and antibiotic uptake, whereas Xiang et al. demonstrated comparable resensitization to cefoperazone-sulbactam in multidrug-resistant and carbapenem-resistant strains when glutamine was supplied. Aminoglycoside uptake depends on the proton motive force, providing a coherent mechanistic basis for the convergent effects of carbon and nitrogen metabolites on resensitization. Together, these studies reinforce the view that simple amino acids can serve as low-toxicity metabolic adjuvants that can shift resistant bacteria into antibiotic-susceptible states (Peng et al., 2015), recasting bacterial metabolism not as a passive physiological background but as a vulnerable target that can be exploited to restore antimicrobial efficacy.

In addition to metabolic adjustments, bacterial persistence and tolerance continue to pose significant challenges to the effectiveness of antimicrobial therapy. Persister cells, the antibiotic-tolerant subpopulation behind chronic and relapsing infections (Lewis, 2010), were examined by Liu et al. In meropenem-exposed *Acinetobacter baumannii*, they identified marked upregulation of the outer membrane protein OmpW and an unexpected gain in virulence in persister-derived cells, arguing that persistence and pathogenicity are coupled rather than independent traits. This observation is clinically significant because *A. baumannii* is among the WHO critical-priority pathogens, and persistence has long been treated as a stalling tactic rather than a virulence asset. OmpW emerges as a candidate target for adjunctive therapies and as a potential biomarker for cells that have entered the tolerant state.

Another major challenge highlighted in this Research Topic is the intrinsic resistance mechanisms of gram-negative pathogens. The outer membrane remains the most challenging permeability barrier in antibacterial therapy, and gram-negative bacteria continue to dominate the critical-priority tier of the 2024 WHO Bacterial Priority Pathogens List (World Health Organization, 2024).

Xu et al. review current strategies targeting this barrier, including direct membrane disruption, inhibitors of lipopolysaccharide biosynthesis and efflux pumps, siderophore-conjugated antibiotics, and nanoparticle-based delivery systems. This review pays particular attention to a new generation of peptide antibiotics, including darobactin and xenorceptides, which exploit the β-barrel assembly machinery responsible for outer membrane protein folding (Imai et al., 2019), a target absent from human cells with limited cross-resistance reported so far.

Probiotics and their secreted metabolites form a complementary therapeutic axis. Tenea et al. characterized *Lactiplantibacillus plantarum* UTNGt28L from Amazonian star apple, combining whole-genome sequencing with metabolomics to identify phenyllactic acid and γ-aminobutyric acid among its antimicrobial outputs, while confirming its safety and probiotic credentials. Bui et al. review the broader antagonism between *L. plantarum* and *Staphylococcus aureus*, covering bacteriocin production, biofilm disruption, quorum-sensing interference, and host immune modulation. Piras et al. bring these concepts closer to the clinic: cell-free supernatants from *Lacticaseibacillus rhamnosus* retained anti-*P. aeruginosa* activity in an air-liquid interface lung model and remained stable in formulations compatible with inhalation delivery, a meaningful step toward postbiotic respiratory therapeutics. Cell-free preparations also bypass the regulatory and safety questions of delivering live organisms to compromised hosts, which has helped postbiotics gain popularity as a tractable translational format. Together, these studies broaden the therapeutic landscape of microbial metabolites beyond direct antimicrobial activity toward microbiome-mediated protection, immune modulation, and translational development.

Two additional contributions extend the scope beyond direct bacterial killing. Zhao et al. reported that indole-3-propionic acid, a gut microbial metabolite, reduces inflammation and restores the blood-milk barrier in *S. aureus*-induced mastitis by activating the aryl hydrocarbon receptor, illustrating that microbial metabolites can act on the host as well as the pathogen and aligning with the growing field of host-directed antimicrobial therapy. Wang et al. synthesized quaternary ammonium salt agents and developed antibacterial coatings for polypropylene surfaces, with activity against biofilm-forming pathogens, antioxidant and anti-inflammatory effects, and acceptable biocompatibility—features relevant to indwelling medical devices and high-touch healthcare environments, where biofilm-mediated colonization remains a leading cause of treatment failure.

Taken together, the contributions to this Research Topic argue for a broader conception of the functions of bacterial metabolites. Rather than serving only as bactericidal scaffolds, they emerge as modulators of resistance proteins, levers on bacterial central metabolism, disruptors of virulence and persistence, and agents that engage host immunity and tissue repair. The Research Topic also reflects how genome mining, metabolomics, molecular dynamics, and machine learning are now standard tools in antimicrobial discovery, and how translational formats such as inhaled postbiotics, antimicrobial coatings, and amino acid adjuvants are becoming part of the conversation. However, important gaps remain: *in vivo* efficacy data are still scarce for several of the proposed strategies; pharmacokinetics, formulation stability, and scalable biosynthesis are not yet routinely addressed; the resistance dynamics of metabolic adjuvants and host-directed agents are largely unstudied; and the regulatory pathway for postbiotic preparations is still poorly defined. We hope this Research Topic helps frame the next steps and supports continued work across natural product chemistry, microbiology, computational biology, and clinical practice.
